# Irish Books and English Reviewers

**Published:** 1911-02-18

**Authors:** 


### The Hospital

A Journal of

The Medical Sciences and Hospital Administration.

New Series. No. 211, Vol. VIII [No. 1283, Vol XLIX.] SATURDAY, FEBRUARY 18, 1911.

### Irish Books and English Reviewers.

If the quarrel which has arisen between a well-known Irish obstetrician and the Journal Committee of the British Medical Association involved simply and solely a difference of opinion as to the merits of a recent book by another Dublin midwifery specialist, it would be savouring of intrusion or impertinence to allude to it in these quite neutral pages. As a matter of fact, however, it raises several important questions of principle connected with the reviewing of medical books, and demands rather more than the indifference of the casual onlooker of a personal dispute. In a review of one of his books, which appeared in the *British Medical Journal* some time ago, Dr. Jellett found passages of criticism to which he strongly objected. The (anonymous) author of that review seemed to him a very long way behind the times, obstetrically speaking; and Dr. Jellett took the obvious course of submitting no more books to that paper. He noticed, however, reviews of other books on obstetrics and gynæcology written in the same carping spirit, and betraying the same prejudice against modern advances. Finally, he read a review of a book by a Dublin colleague which seemed so scandalous that he could keep silent no longer. Accordingly he wrote to the editor protesting against "such gross travesties of modern criticism," and against an opinion "which condemns or praises according to the fads of the reviewer and to the geographical position of the reviewed." The editor refused to publish the letter, on the ground that it contained a personal attack on the reviewer. To this Dr. Jellett replied, offering to delete the offending paragraph. This offer was also refused, but unfortunately there ensued a delay of nearly four months before the confirmatory decision of the committee was conveyed to Dr. Jellett, a delay which certainly seems a reasonable ground for complaint. As the Dublin obstetrician could not obtain publicity for his grievance in the *British Medical Journal*, he has sent a copy of his letter, together with a covering letter of explanation, to the *Medical Press*.

The facts of the case as here presented are, of course, an *ex parte* statement; and there may be another side to the question. But, taking Dr. Jellett's case as he sets it out, it would seem that he has got a genuine grievance. His letter is bitter in tone, certainly, but the editor of the *British Medical Journal* is either unduly sensitive to criticism or else hyper-loyal to his reviewer in refusing to publish it. That the references are sufficiently pointed to reveal the identity of the particular reviewer may perhaps have weighed with him, but it is difficult to see any great hardship in that, in these days when the signed, or at least initialled, review is steadily coming into favour. It is equally hard to resist the conclusion that Dr. Jellett has some right on his side when he levels an accusation of antiquated prejudice against the *Journal's* reviewer.

Setting aside these personalities, it may be noted that the reviewing of medical books is an art which differs in certain respects from that of reviewers for lay journals. Fairmindedness, a logical faculty, culture and wide reading, detachment, sympathy, and a capacity for expressing his conclusions: all these are qualities which every reviewer ought to have. The medical reviewer should be selected for these qualifications, doubtless, but there are others which, in his case, are perhaps as important. Foremost is knowledge, by which is meant a working knowledge of the subject treated of in the book reviewed. It is useless to set a bacteriologist to review a book on radiology, for instance. Text-books on branches of medical science are written for the most part by specialists; necessarily so, as a general rule. Need, then, the reviewer also be a specialist in the same branch of study? Preferably, but not necessarily so, it may be said. Then again, the reviewer has to bear in mind the circle to whom the author is addressing himself. A specialist writing of his specialty for students must adopt a different line from one compiling an advanced and cyclopædic treatise for his own fellow specialists. So also must the reviewer bear in mind these limitations; he must not quarrel with an author for being too dogmatic in a book for students, too practical in one for general practitioners, of too profound and ingenious in one for specialists. Nor must the medical reviewer be too captious over small points; opinions differ over many points still, especially matters of detail, and even actual errors are often better overlooked. If the work be honestly done, be likely to suit the needs of those for whom it is designed, be in the main sound and trustworthy—that is a book worthy of praise in a medical journal, however much the opinions of the reviewer may differ from those of the author on various points. On the other hand, for slipshod books, overloaded with quotations from the writings of others, and leaving the reader in doubt as to the convictions of the writer on important questions, there should be sharp condemnation without fear or favour. No consideration of the advertisement orders of the publishing firm sending the book should be allowed to weigh for a moment either way. Lastly, medical reviewers have always to remember that the authors of text-books are in many cases quite unpractised in English composition, which may be distasteful to them. If a man can express his thoughts in scholarly style, so much the better for all concerned. But if he lacks this power it does not follow that his book need thereby stand condemned. Without excusing the slovenly stuff that issues from medical publishing houses not infrequently, it is yet true that style is less important in medical literature than in other branches of that art.

